# Method for Estimating Temporal Gait Parameters Concerning Bilateral Lower Limbs of Healthy Subjects Using a Single In-Shoe Motion Sensor through a Gait Event Detection Approach

**DOI:** 10.3390/s22010351

**Published:** 2022-01-04

**Authors:** Chenhui Huang, Kenichiro Fukushi, Zhenwei Wang, Fumiyuki Nihey, Hiroshi Kajitani, Kentaro Nakahara

**Affiliations:** Biometrics Research Labs, NEC Corporation, Abiko 1131, Japan; k-fukushi@nec.com (K.F.); w-zhenwei@nec.com (Z.W.); nihey@nec.com (F.N.); h-kajitani@nec.com (H.K.); k-nakahara@nec.com (K.N.)

**Keywords:** gait parameters assessment, gait event detection, foot motion, gait analysis, in-shoe sensor, inertial measurement unit

## Abstract

To expand the potential use of in-shoe motion sensors (IMSs) in daily healthcare or activity monitoring applications for healthy subjects, we propose a real-time temporal estimation method for gait parameters concerning bilateral lower limbs (GPBLLs) that uses a single IMS and is based on a gait event detection approach. To validate the established methods, data from 26 participants recorded by an IMS and a reference 3D motion analysis system were compared. The agreement between the proposed method and the reference system was evaluated by the intraclass correlation coefficient (ICC). The results showed that, by averaging over five continuous effective strides, all time parameters achieved precisions of no more than 30 ms and agreement at the “excellent” level, and the symmetry indexes of the stride time and stance phase time achieved precisions of 1.0% and 3.0%, respectively, and agreement at the “good” level. These results suggest our method is effective and shows promise for wide use in many daily healthcare or activity monitoring applications for healthy subjects.

## 1. Introduction

As a result of the rapid development of wearable device technologies, wearable smart motion sensors have been used in various healthcare applications based on daily gait analysis, with all data processing performed automatically on an edge device [1]. Recently, a new type of wearable smart motion sensor for daily gait analysis, called an “in-shoe motion sensor” (IMS), was developed. The IMS can be placed in various kinds of shoes or insoles, and is less inconvenient to wear [2,3]. Thus, it shows promise for various healthcare and activity monitoring applications, with the aim of improving the habits of healthy subjects involving daily walking, by assessing temporal gait parameters or detecting gait signal features through artificial intelligent technologies [4]. 

Common temporal gait parameters such as walking velocity, stride length, foot angle, gait variability, cadence, and foot clearance can currently be obtained in real time by automatic calculation in the microprocessor of an IMS [4,5]. In contrast, temporal gait parameters concerning bilateral lower limbs (GPBLLs) play more important roles in healthcare or daily activity monitoring applications, such as evaluation of walking ability [4], metabolic evaluation [6], daily fatigue monitoring [7], and alcohol use monitoring [8]. These parameters include the double support time (DST), which is defined as the duration when both bilateral lower limbs touch the ground in one gait cycle (GC), and the symmetry index of stride time (*SIS_tr_*) and symmetry index of stance phase time (*SIS_ta_*) between the two limbs. These GPBLLs are important because they capture the interaction of the two feet and are essential predictors for estimating certain deep gait parameters, such as gait asymmetry, lower limb muscle strength, and muscle strength transition ability from one limb to the other [5,6,9]. Furthermore, these deep gait parameters are significant predictors for metabolic monitoring, fatigue assessment, walking ability, body functions, and alcohol monitoring [5,6,7,9,10,11,12]. To expand the potential use of IMSs in healthcare or daily activity monitoring applications for healthy subjects, we believe it is necessary to conduct automatic measurement of these GPBLLs by an IMS. 

**Figure 1:**
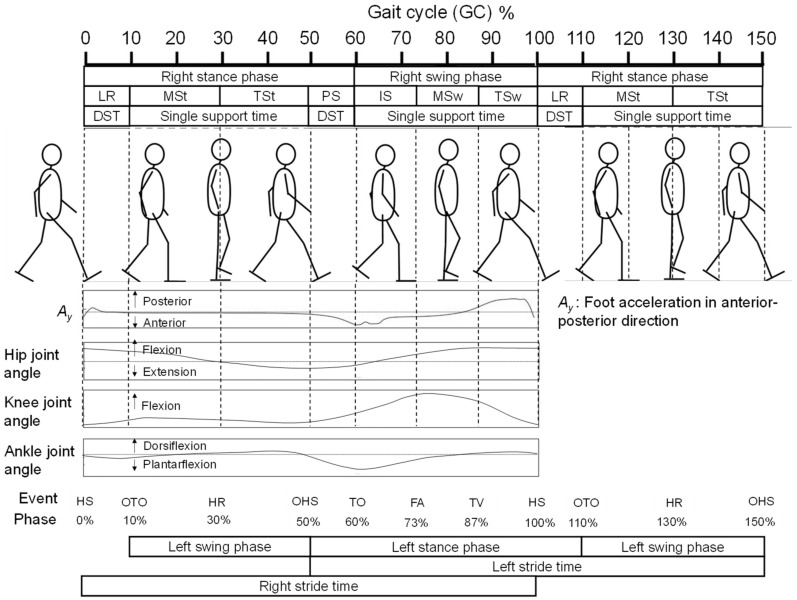
Illustration of the definitions of a GC, gait phases, gait events, and certain temporal gait parameters, and information on the foot acceleration in the anterior–posterior direction, the knee joint angle, and the ankle joint angle in one GC.

To obtain the temporal GPBLLs, which are illustrated in Figure 1, it is necessary for foot motion signals to interact well with each other. The motions of both feet are commonly detected by placing two independent sensors on them. Measurement then requires the phase difference between the motions in the same GC to be correctly traced on the same temporal axis. A common approach is to temporally synchronize the sensors using external devices, i.e., via a hardware connection [13,14]. In a smart system, the IMSs are synchronized with each other by timestamps that originate from a universal time controller in the system, e.g., a smartphone. This may make the system more complicated and subject to the uncertainty in the sensor network, clock drift, and packet delays in the communication protocol [15,16].

Accordingly, in this study, by combining biomechanical knowledge with signal processing, we propose a new approach to overcome these issues and provide a simpler method with higher accuracy and precision for evaluation of temporal GPBLLs using only a single sensor. Walking is a natural form of periodic movement. Moreover, during walking, the motions of the two lower limbs intrinsically interact well with each other, according to a musculoskeletal model in which the phase difference between the two limbs is locked by the connection of the pelvis. Thus, once the motion of one foot is determined, the motion of the opposite foot can also be known almost immediately [17].

A GC is the time period or sequence of events or movements during locomotion from when a foot contacts the ground, which is called a “heel strike” (HS), to when that same foot again contacts the ground. The HS is one of seven defined gait events, i.e., special motions during walking. A GC can be partitioned into stance and swing phases, which constitute 60% and 40% of the GC, respectively, as shown in Figure 1. Furthermore, a GC can be divided into seven periods—loading response (LR), mid-stance (MSt), terminal stance (TSt), pre-swing (PS), initial swing (ISw), mid-swing (MSw), and terminal swing (TSw)—by the six gait events in addition to HS: opposite toe off (OTO), heel rise (HR), opposite heel strike (OHS), toe off (TO), feet adjacent (FA), and tibia vertical (TV) [17]. Specifically, taking the right foot as an example, the OHS and OTO events represent the respective moments when the left heel strikes the ground and the left toe departs from the ground, which suggests that the left foot’s exact temporal information for GPBLL estimation can be obtained by detecting these two gait events from the right foot. Hence, as long as the HS, TO, OHS, and OTO events of the right foot are appropriately detected from foot motion signals, temporal GPBLLs such as DST, *SIS_tr_*, and *SIS_ta_* can be captured well by a single IMS mounted on the right foot. In this case, hardware connection of two sensors would no longer be necessary to assess the interaction of two feet. We refer to this idea as the “gait event detection approach”, which requires an IMS to have a real-time gait event detection method.

In our previous study, we developed a rule-based real-time HS and TO detection method [18]. As shown in Figure 1, we discovered that the foot acceleration signal in the anterior–posterior direction exhibits a sharp valley next to the maximum, which can be used for HS detection, while the first valley in a W-shaped wave pattern appearing after a foot-flat state can be used for TO detection. The missing piece of this approach is a method of real-time OHS and OTO event detection for an IMS. Currently, many gait event detection methods for wearable sensor systems have been proposed [19,20]. Previously, González et al. [21] proposed a method using a foot pressure sensor, and Liu et al. [22] proposed a method based on fusion of data from multiple segments of the lower limbs to detect OHS and OTO. However, these methods cannot be used in an IMS system that only measures a single segment. For OHS and OTO detection using foot-mounted inertial measurement units (IMUs), Mariani et al. [23] developed hidden Markov models, and Kidziński et al. [24] proposed a deep learning method. Such methods that construct complicated models from training data require considerable processing power and, thus, they are difficult to implement in a microprocessor chip. In contrast, Rueterbories et al. [25] developed a simpler, rule-based OHS and OTO detection method, which was considered feasible for edge device processing. In their method, they determine gait events through detection of peaks and valleys from three types of low-pass-filtered composite signals, including a raw signal at 160 Hz and two moving-average raw signals with window lengths of 50 and 200; then, they analyze the relationships between detected feature points. However, their method was sensitive to individual walking velocity differences: when the deviation of the velocities from the average increased, the precision of estimation decreased. To achieve higher precision, improved OHS and OTO detection methods are thus needed.

According to modern kinesiology studies on walking, gait events correspond to the timings of muscle and joint activity patterns transiting from one state to another [17,26]. Following these studies, we hypothesize that gait events should appear as characteristic turning points, such as peaks, valleys, and zero-crossing points, in the foot motion signal acquired by an IMS. In the current study, we developed a rule-based method for OTO and OHS detection and applied it to GPBLL measurements. To do so, we first used biomechanical knowledge to analyze how the lower limbs move near the moments of OHS and OTO [17], and we then searched for possible feature points as candidates for OHS and OTO detection based on the aforementioned hypothesis. Next, we evaluated the accuracy and precision of our method through comparison with the measurement results obtained by a 3D motion analysis system. Given the similarity in motion between the left and right feet, if the feasibility of our method is demonstrated by an IMS mounted on the right foot, we assume that it should also be feasible for an IMS mounted on the left foot.

## 2. Materials and Methods

### 2.1. Participants

In this study, we recruited 26 participants, including 20 males and 6 females, and collected data including their gender, age, height, weight, and shoe size. The average age was 39.3 ± 9.5 years, the average height was 169.5 ± 7.7 cm, the average weight was 67.2 ± 12.1 kg, and the average shoe size was 26.4 ± 1.0 cm. All subjects could walk independently without any assistive device such as a cane, crutch, or orthotic device. Participants had normal or corrected-to-normal vision, no history of neuromuscular or orthopedic diseases, and no obstacles in communication. The experimental procedure was explained to all subjects, and informed consent was obtained individually before the experiment. The study was approved by the NEC Ethical Review Committee for the Life Sciences (protocol number: LS2019-008) on 24 July 2019.

### 2.2. Experimental Setup

**Figure 2:**
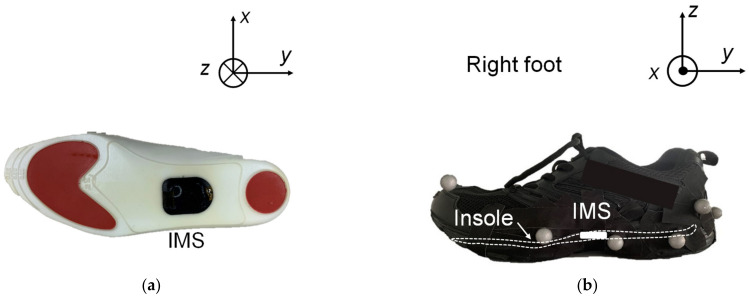

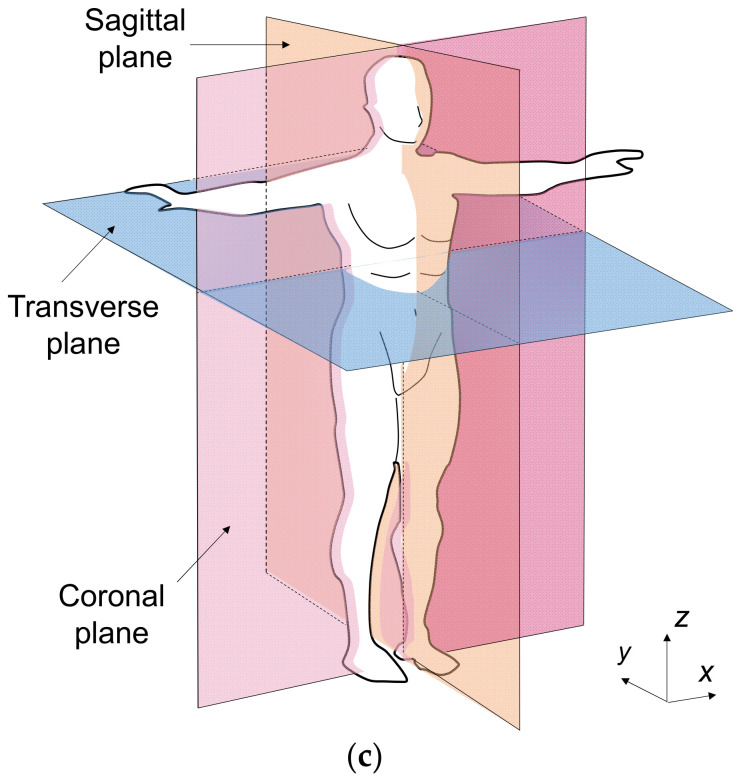
(**a**) Schematic of an IMS embedded in an insole. (**b**) Schematic of the insole inserted in an athletic shoe. (**c**) Definition of the coordinate axes and corresponding planes.

Figure 2 illustrates the experimental environment and setup. To prevent discomfort during walking, the IMS was mounted in an insole that was placed under the foot arch. We considered that, in practical use, compared with smart sensors fixed in/on the shoe, this would allow the user to insert the insole into the shoes they preferred. The size of the IMS was 3.0 × 3.5 × 0.6 cm^3^. (Figure 2a). It included a 6-axis IMU, microprocessor, Bluetooth module, and control circuit. We assumed that the midfoot–hindfoot was a rigid body and that the shoe fit tightly on the foot, so that the signal from the IMS could be treated as the foot-motion signal. To verify the feasibility of the proposed estimation method, we mounted the IMS on the right foot to estimate the motion of the left foot (Figure 2b). The IMS signal comprised nine types of signal: the three directly measured axes of acceleration, *A_x_* (medial: +; lateral: −), *A_y_* (posterior: +; anterior: −), and *A_z_* (superior: +; inferior: −); the three angular velocity components, *G_x_* (plantarflexion: +; dorsiflexion: −), *G_y_* (eversion: +, inversion: −), and *G_z_* (adduction: +, abduction: −); and the three sole-to-ground angles (SGAs), namely, the roll (*E_x_*), pitch (*E_y_*), and yaw (*E_z_*). The SGA directions were defined in the same way as for the angular velocity, and the SGAs were calculated internally using a Madgwick filter [27]. The acceleration values from the IMS signal were corrected to inertial coordinates (Figure 2c).

A 3D motion analysis system, the Track 3 (Vicon Motion Systems, Oxford, UK), was used to obtain reference measurements. The motion analysis space was partitioned by 10 Bonita B10 motion-capture cameras (Vicon Motion Systems, Oxford, UK), with five cameras on each side of a straight path. Optical reflection markers were attached to the surfaces of the two shoes. One marker was located at the toe, and the others were at the midfoot and hindfoot (Figure 2b). The markers on the other shoe were attached symmetrically. The midfoot and hindfoot markers were combined to represent a rigid body whose center of gravity was at the heel. As a result, first, all the SGAs could be represented by the Euler angles of the rigid body; second, the trajectory of the rigid body was equivalent to that of the heel, so that the minimum of the trajectory of one stride specified the timestamp of an HS (*T_HS_*). The traced trajectory of the toe marker was used to specify the toe’s movement, so that the minimum of the trajectory specified the timestamp of a TO (*T_TO_*) event. Finally, the timestamps of the minima of the left heel trajectory (*T_OHS_*) and left toe trajectory (*T_OTO_*) were treated as the reference timestamps for OHS and OTO detection.

### 2.3. Experimental Protocol

The participants were asked to walk at a self-determined, comfortable speed in an 8 m straight line along a pathway about 0.8 m wide, for four successive trials. Before data collection, the participants were given a 2 min practice session to familiarize themselves with the environment and procedure. The data sampling frequency (*f_s_*) was set to 100 Hz for both the IMS and Vicon measurement systems. The measurement range was ±16 g for acceleration and ±2000°/s for angular velocity in the IMS.

### 2.4. Feature Selection for OHS and OTO Detection from Foot Motion through Biomechanical Analysis

**Figure 3:**
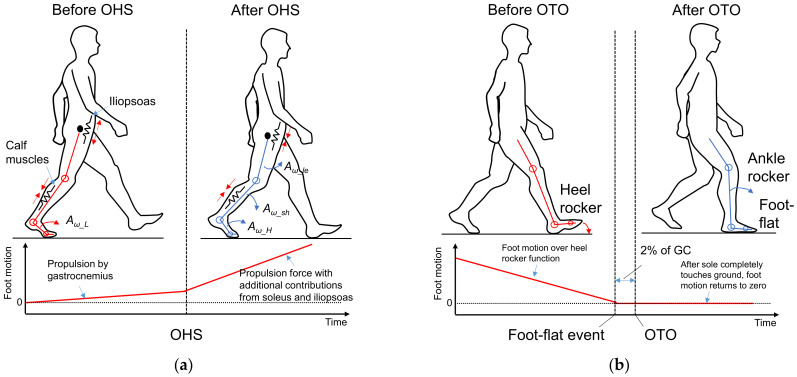
Foot motion analysis based on standard kinesiology knowledge: (**a**) foot motion near OHS, and (**b**) foot motion near OTO.

Because walking motions occur in the anterior–posterior direction, we mainly focused on motion of the lower limbs in the sagittal plane. Figure 3 shows a schematic of the motions of the right shank and foot before and after OTO and OHS gait events, in reference to alterations of the hip, knee, and ankle joints in one GC [17] (also see Figure 1). Through analysis based on the biomechanical knowledge explained in this section, we determined how to select candidate feature points from the IMS signal.

#### 2.4.1. Foot Motion near OHS

According to standard biomechanical knowledge [17,28], as shown in Figure 3a, during TSt, i.e., before the OHS event, force is generated in preparation for forward limb propulsion to accelerate the foot motion. Most of this force is supplied by concentric activity of the gastrocnemius muscle, an important part of the calf muscles, which causes the ankle to enter plantarflexion as the heel rises off the floor with the forefoot rocker, while the left foot remains on the floor [28,29]. The hip and knee remain extended to support the body when the heel leaves the ground. The hip flexor, mainly the iliopsoas, is stretched and thus stores energy like a spring. In this phase, the changes in the knee and ankle joint angles are small. We can thus assume that this part of foot motion is performed as low-rotation motion (*A_ω_L_*). 

Then, after entering PS, i.e., after OHS, once the left heel strikes the ground, the body also obtains support from the left foot. The propulsive force continues during PS with additional contributions from the soleus, another important part of the calf muscles, and the iliopsoas. The energy stored in the iliopsoas starts to be released, and consequently, the hip starts to enter flexion to prepare for swinging, while the foot progression continues over the forefoot rocker. Knee flexion results passively from the combined motions of hip flexion and ankle plantarflexion [28]. According to a model of the alignment of joints and segments [29], the leg’s momentum is transferred through the knee joint and then causes shank rotation (*A_ω_sh_*) in the anterior direction, which is appended to the foot through the ankle joint. Combined with the existing foot rotation, the foot’s total rotation motion suddenly becomes larger, and we can thus assume that this part of foot motion shifts to high-rotation motion (*A_ω_H_*) during PS. 

Based on the assumptions mentioned above, we hypothesized that at OHS, there should be a sudden gradient alteration in the foot motion in the sagittal plane, which is induced by the sudden increases in the absolute signal values between 40% of the GC and *T_TO_*. Here, we refer to the points where the signal gradient suddenly changes as “gradient turning points” (GTPs), and we decided to use these GTPs in the foot motion signals in the sagittal plane, i.e., *A_y_*, *A_z_*, and *G_x_*, as the candidate feature points for OHS detection.

#### 2.4.2. Foot Motion near OTO

As shown in Figure 3b, the musculoskeletal model [17,28] reveals that from the right HS, the trunk moves toward the right foot until the body is erect, with only the right limb supporting the whole body along with the heel rocker. As shown in Figure 3b, before OTO, the body is supported by both lower limbs, which allows the right foot to rotate and finish the heel rocker motion; in contrast, after OTO, only the right foot supports the whole body, which means that it must be flat to maintain balance. According to the traditional subdivision of the gait cycle, a foot-flat event occurs at around 8% of the GC, whereas in the modern subdivision of the gait cycle, OTO occurs at around 10% of the GC [17]. Hence, as long as a foot-flat event is detected, OTO can also be detected by appending this 2% difference. It is known that the foot motion signal almost returns to zero after the sole completely touches the ground. We hypothesized that at a foot-flat event there should be GTPs induced by the foot motion signal returning to zero after *T_HS_* in the sagittal plane. Accordingly, we decided to use these GTPs in the foot motion signals, *A_y_*, *A_z_*, and *G_x_*, as the candidate feature points for OTO detection.

### 2.5. Data Processing for Analysis

**Figure 4:**
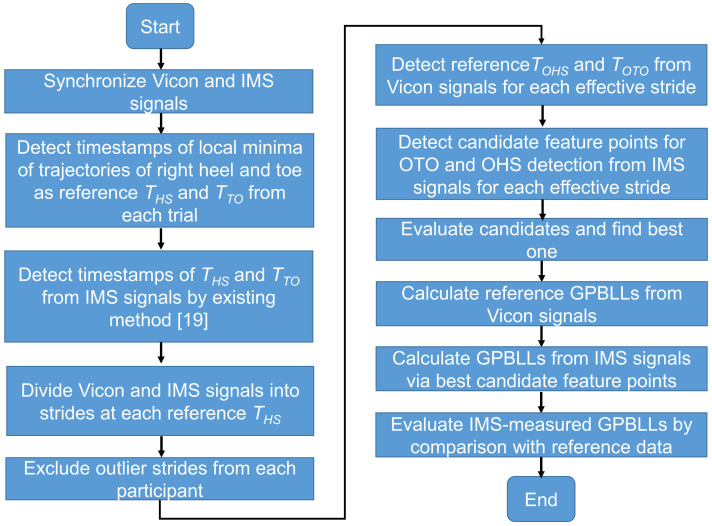
Flowchart of the signal processing procedure in this study.

Figure 4 shows the signal processing procedure used for real-time OHS and OTO detection in this study. 

To identify which candidate point is the best for OHS and OTO detection and for the evaluation of the proposed GPBLL estimation method, we first needed to completely synchronize the signals from the Vicon system and the IMS (details in Supplementary Materials). All the data processing and analysis were performed in MATLAB (Mathworks, Natick, MA, USA).

**Figure 5:**
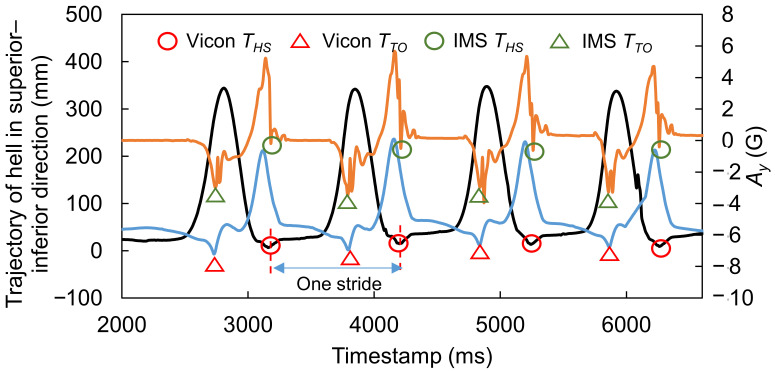
Example showing how to divide data into strides. Black and blue curves represent the trajectories of the heel and toe in the *Z* direction, and the orange curve represents IMS signal *A_y_*.

After synchronization of each trial, we obtained a result like the example shown in Figure 5. Here, the reference *T_HS_* and *T_TO_* values could be detected by searching for local minima in the trajectories of the heel and toe in the *Z* direction (black and blue curves, respectively), while the HS and TO timestamps in the IMS signal *A_y_* (orange curve) could be detected by the peak detection algorithm described in our previous study [18]. Then, the synchronized data were divided into strides at two reference *T_HS_* points. We originally obtained a total of 679 strides. Before applying the OHS and OTO detection methods, the first and last two strides of each trial were excluded. Furthermore, some strides existed in the dataset that had a different waveform shape, which was probably induced by, for example, sensor error or accidental events disturbing the walking motion. These outliers may need to be removed from the dataset in order to retain the strides that can represent the true gait characteristics of participants [30] from the remaining strides. We removed these outliers, leaving a total of 342 effective strides that were recorded.

In the next step, we recorded the reference *T_OHS_* and *T_OTO_* from the trajectories measured by the Vicon system, and we obtained all the candidate points mentioned in Section 2.4.1 from each effective stride. Because we did not previously know which candidate point was the best for OHS and OTO detection, before calculating GPBLLs in the final step, the candidate whose timestamp had the best synchronicity and agreement with the reference was judged as the best one, by comparing the synchronicities between the candidate points and the reference data. Then, the best candidates in the IMS signals were chosen for constructing OHS and OTO detection algorithms. In the final step, the GPBLLs were calculated from the Vicon and IMS signals as reference and estimated values, respectively, and they were compared to evaluate the effectiveness of our proposed method.

### 2.6. Candidate Feature Points for OHS and OTO Detection

In this section, we explain the acquisition of candidate feature points from an IMS signal. The OHS and OTO detection algorithm is customized according to the characteristics of the best candidates.

**Figure 6:**
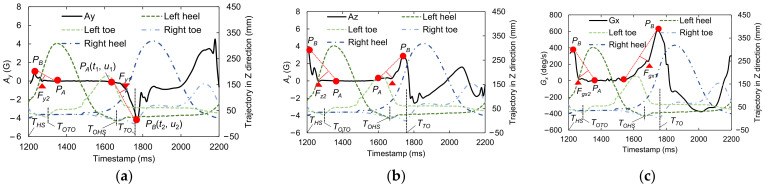
Examples of the foot motion signals, (**a**) *A_y_*, (**b**) *A_z_*, and (**c**) *G_x_*, with candidate feature points for OHS and OTO detection and the marker trajectories in the superior−inferior (*Z*) direction on the left and right heels and toes.

Figure 6 shows examples of the foot motion signals, *A_y_*, *A_z_*, and *G_x_*, and the trajectories of the markers in the superior–inferior (*Z*) direction on the left and right heels and toes, which contain all the information in one GC. In this case, HS occurred around 1200 ms and TO occurred around 1720 ms. The aforementioned candidate feature points for OHS and OTO detection are indicated by red triangles. These GTPs are detected from the waveform via the triangle thresholding algorithm (TTA) [31]. Please note that the TTA is applied independently on each signal from the sensor and then validated using the Vicon’s measurement. We use this algorithm because, if the reference points (*P_A_* and *P_B_* in the figure) are appropriately chosen, a partial waveform in the region of interest can be treated as a monotonic curve without inflection points when tiny fluctuations are ignored. This type of curve is then suitable for application of the TTA.

For OHS detection, *P_A_* can be selected at the moment when the amplitude of the foot motion signal exceeds the baseline level in a foot-flat state before TO, and *P_B_* can be selected at the local maximum (or minimum for *A_y_*) before TO. For OTO detection, *P_A_* can be selected at 20% of the GC after HS, because most people are in a foot-flat state at that time, and *P_B_* can be selected at the nearest local maximum to 20% of the GC after HS. Here, to obtain the length of a GC, the duration between an HS and the previous HS is also recorded. HS and TO event detection in the IMS signal is performed by the peak detection algorithm in our previous study [19].

To apply the TTA to a waveform, we first extract an appropriate set of reference points, *P_A_*(*t*_1_, *u*_1_) and *P_B_*(*t*_2_, *u*_2_), which are located in front of and behind the target point, respectively. Then, a straight line *U*(*T*) passing through *P_A_* and *P_B_* is constructed via Equation (1):

(1) $$U\left(T\right)=\left(\frac{u_{2}-u_{1}}{t_{2}-t_{1}}\right)\left(T-t_{1}\right)+u_{1}.$$

The expected GTP, *T_GTP_*, is the furthest point on the curve of interest, *Q*(*T*), which is the partial waveform between *t*_1_ and *t*_2_. The distance *D*(*T*) between the points on *Q*(*T*) and the line *U*(*T*) is expressed by Equation (2), and the GTP is then obtained by Equation (3):

(2) $$D\left(T\right)=\frac{\left|\left(\frac{u_{2}-u_{1}}{t_{2}-t_{1}}\right)\left(T-t_{1}\right)+u_{1}-Q\left(T\right)\right|}{\sqrt{1+{\left(\frac{u_{2}-u_{1}}{t_{2}-t_{1}}\right)}^{2}}},$$

(3) $$T_{GTP}=\underset{t_{1}\leq T\leq t_{2}}{\mathrm{argmax}}D\left(T\right).$$

**Figure 7:**
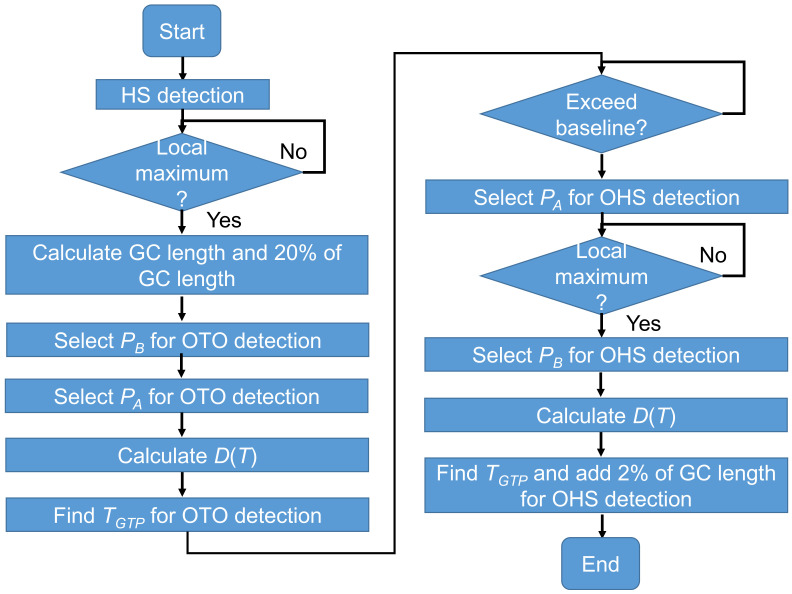
Algorithm for OHS and OTO detection in one GC from the IMS signal.

Figure 7 shows a flowchart of OHS and OTO detection in one GC via our rule-based gait event detection algorithm for IMS signals. This algorithm is feasible for installation on an edge device. When the data stream arrives, the HS is first detected to specify the start of one step. Next, according to the timestamp of the previous HS, the length of one GC and 20% of that GC are calculated. Here, for OTO detection, the nearest local maximum to 20% of the GC is selected as *P_B_*, and the 20% location is selected as *P_A_*. The waveform between *P_A_* and *P_B_* is sent to a buffer, and *T_GTP_* for OTO detection is then obtained from the calculated *D*(*T*). Next, within one GC after entering a foot-flat state, the data stream is monitored to check whether it exceeds the baseline, as judged by a threshold. In this study, the thresholds for *A_y_*, *A_z_*, and *G_x_* were 0.15 g, 1.15 g, and 30 deg/s, respectively, which were considered higher than the noise levels. For OHS detection, the point exceeding the threshold is selected as *P_A_*, and another local maximum is selected as *P_B_*. Finally, *T_GTP_* for OHS detection is detected by the TTA.

To test the agreement between the IMS and Vicon measurements in all strides, we first set a reference point, *T_HS_ref_*, indicating the true HS timestamp in every stride as determined by the Vicon system. Then, we express the Vicon and IMS measurements in terms of the relative OHS and OTO time in one stride, *T_OHS__*_0_ and *T_OTO__*_0_, via Equations (4) and (5):

(4) $$T_{OHS\_0}=T_{OHS}-T_{HS\_ref},$$

(5) $$T_{OTO\_0}=T_{OTO}-T_{HS\_ref},$$

where *T_OHS_* and *T_OTO_* are the OHS and OTO timestamps in the data stream, respectively.

### 2.7. Temporal GPBLL Estimation through Gait Event Detection Approach

As long as HS, TO, OHS, and OTO in one stride are detected through the flow shown in Figure 5, temporal GPBLL estimation can be performed as follows.

As shown in Figure 1, the DST actually consists of two sub-phases in the stance phase: LR (*DST*_1_) and PS (*DST*_2_). Here, *DST_t_* is defined as the total of *DST*_1_ and *DST*_2_ and can be expressed by Equation (6):

(6) $$DST_{t}=\left(T_{OTO}-T_{HS}\right)+\left(T_{TO}-T_{OHS}\right).$$

The stance phase time (*T_sta_l_*) and stride time (*T_str_l_*) of the left foot and the stance phase time (*T_sta_r_*) and stride time (*T_str_r_*) of the right foot can be expressed by Equations (7)–(10):

(7) $$T_{sta\_L}=T_{OTO}-T_{OHS},$$

(8) $$T_{str\_L}={T^{\prime }}_{OHS}-T_{OHS},$$

(9) $$T_{sta\_R}=T_{TO}-T_{HS},$$

(10) $$T_{str\_R}={T^{\prime }}_{HS}-T_{HS},$$

where *T*’*_HS_* and *T*’*_OHS_* denote the timestamps of HS and OHS in the next stride, respectively. Additionally, the symmetry indexes of the stance phase time (*SIS_ta_*) and the stride time (*SIS_tr_*) can be expressed by Equations (11) and (12), which are modified from the definitions given in [5,11]:

(11) $$SIS_{ta}=\frac{T_{sta\_L}-T_{sta\_R}}{0.5\left(T_{sta\_L}+T_{sta\_R}\right)},$$

(12) $$SIS_{tr}=\frac{T_{str\_L}-T_{str\_R}}{0.5\left(T_{str\_L}+T_{str\_R}\right)}.$$

Here, we retain positive and negative values instead of using absolute values in the calculated results for *SIS_ta_* and *SIS_tr_* to express both the symmetry and the magnitude relationship of the temporal gait parameters between the left and right feet.

Note that the *T_HS_* in Equations (6), (9), and (10) is different from that in Equations (4) and (5). For the Vicon measurements, *T_HS_* was equivalent to *T_HS_ref_*, whereas for the IMS measurements, *T_HS_* was determined from the IMS signal using the peak detection algorithm [18].

### 2.8. Statistical Analysis

**Figure 8:**
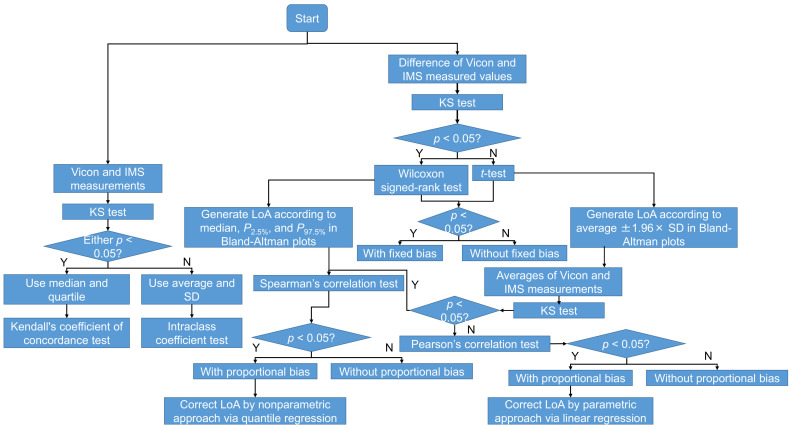
Flowchart of the statistical analysis in this study.

Because the temporal resolution of measurement systems (IMS and Vicon) is 10 ms, the measured data performed as discrete values. To evaluate our method appropriately, we used statistical analysis, whose flowchart is shown in Figure 8. The Kolmogorov–Smirnov (KS) test was used to test the normality of the distributions of the Vicon and IMS measurements of gait event timing, the temporal GPBLLs, and the differences between the measurements. When normality was confirmed, the average and standard deviation (SD) were used; otherwise, the median and quartile were used. The accuracy and precision were respectively expressed as the average and SD or the median and quartile deviation (QD) of the differences between the relative timestamps of gait events and the temporal gait parameters measured by the two systems. In evaluating the gait event detection, the synchronicities of candidate feature points were represented by the average or median of the differences between points.

Bland–Altman plots [32] were used for evaluating the limit of agreement (LoA) between the measurement systems. When the differences followed a normal distribution, the *t*-test was applied to test whether there was a fixed bias between the two systems, and the LoA of the 95% confidence interval was determined from the average difference ±1.96 × SD. Otherwise, the Wilcoxon signed-rank test was applied instead, and the LoA was determined by the 2.5th and 97.5th percentiles (*P*_2.5%_ and *P*_97.5%_) [33]. Next, the significance of the correlation coefficient *r* between the differences (*D*) and averages (*A*) of the two systems’ measurements was used to check for proportional bias. When both the differences and the averages followed a normal distribution, Pearson’s correlation test was applied, and the LoA was corrected by a parametric approach via linear regression, as expressed by Equations (13)–(16) [33]:

(13) $$\tilde{D}=b_{1}A+b_{0},$$

(14) $$R=D-\tilde{D},$$

(15) $$\mathrm{ULoA}=\tilde{D}+1.96\sqrt{\pi /2}R,$$

(16) $$\mathrm{LLoA}=\tilde{D}-1.96\sqrt{\pi /2}R,$$

where $\tilde{D}$ denotes the fitted perfect agreement (PA) line, and *R* denotes the residual between the difference of the two system measurements and the fitted PA line.

When the differences and averages of the systems’ measurements were not both normally distributed, Spearman’s correlation test was applied, and the LoA was corrected by a nonparametric approach via quantile regression [34]. We define (τ) as the τth quantile regression. For each τ, (τ) has a pair of coefficients ($c_{0}^{\tau },c_{1}^{\tau }$) for regression. Then, the PA line, ULoA, and LLoA can be expressed by Equations (17)–(19):

(17) $$\tilde{D}=c_{1}^{50\%}A+c_{0}^{50\%},$$

(18) $$\mathrm{ULoA}=c_{1}^{97.5\%}A+c_{0}^{97.5\%},$$

(19) $$\mathrm{ULoA}=c_{1}^{97.5\%}A+c_{0}^{97.5\%},$$

where ($c_{0}^{2.5\%},c_{1}^{2.5\%}$), ($c_{0}^{50\%},c_{1}^{50\%}$), and ($c_{0}^{97.5\%},c_{1}^{97.5\%}$) correspond to (2.5), *β*(50), and *β*(97.5), respectively. Note that, in this study, *D* was calculated as the IMS measurements minus the Vicon measurements; thus, positive *D* indicates that the IMS values were bigger than the Vicon values, and vice versa.

The agreement between the measurements of the two systems was also assessed. When both measurements followed a normal distribution, intraclass correlation coefficients (ICCs) of types (2, 1) and (2, *k*) were used for evaluating the levels of agreement between one-stride measurements and multiple-stride measurements, respectively. Otherwise, Kendall’s coefficient of concordance (Kendall’s W) was used. The guidelines for interpreting the ICC as an inter-rater agreement measure were as follows: excellent, >0.750; good, 0.600–0.749; fair, 0.400–0.599; and poor, <0.400 [35]. The guidelines for interpreting Kendall’s W as an inter-rater agreement measure were as follows: strong, >0.600); moderate, 0.300–0.599; weak, 0.100–0.299; and almost no agreement, <0.100 [36]. The level of significance was *p* < 0.05 for all tests.

## 3. Results

### 3.1. Evaluation of Signal Features for Gait Event Detection

**Figure 9:**
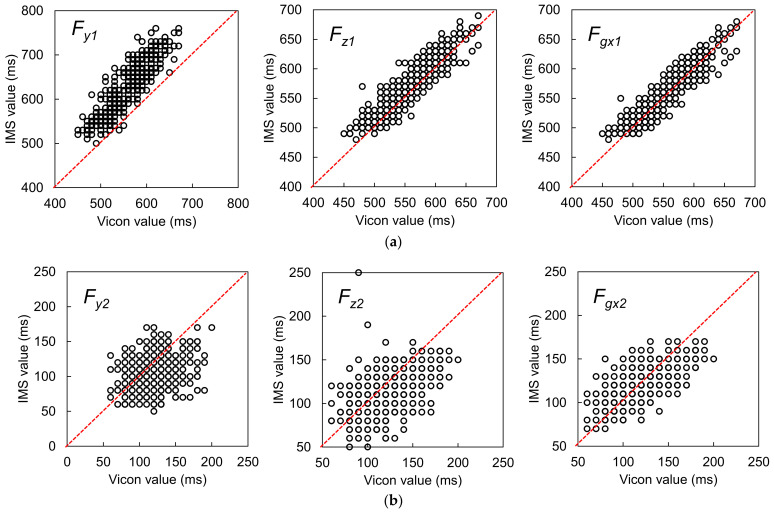
(**a**) Agreement plots for the candidate features for OHS detection, and (**b**) agreement plots for the candidate features for OTO detection. The red dotted lines represent the PA lines.

**Figure 10:**
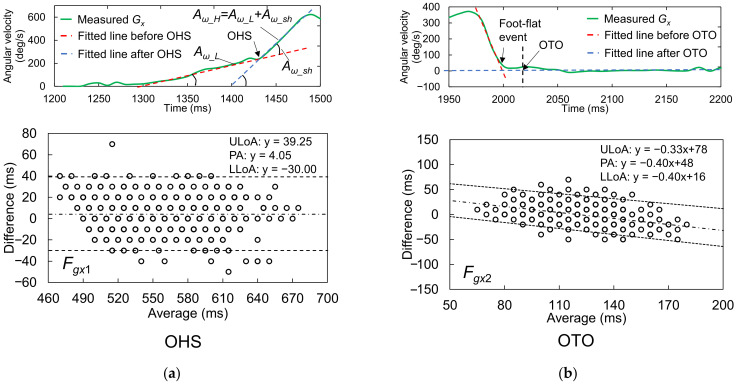
Detailed characteristics and Bland–Altman plots of the best candidates for (**a**) OHS and (**b**) OTO detection. The upper and lower LoAs (ULoA and LLoA, black dotted lines) are shown around the PA (black dashed line) for comparison of the IMS and Vicon gait event detection results. The LoAs for OTO were corrected by a nonparametric approach via quantile regression.

For all candidate features, the measurements and the differences in the measurements between the two systems were judged as not following a normal distribution. Accordingly, the median and quartiles were used for analysis, and the Wilcoxon signed-rank test was applied to check the fixed bias between the two systems. The results are summarized in Table 1, and agreement plots for all the candidate features are shown in Figure 9. The detailed characteristics and Bland–Altman plots of the best candidates for OHS and OTO detection are shown in Figure 10. As described above, the LoAs were determined by *P*_2.5%_ and *P*_97.5%_. Note that, because the temporal resolution was 10 ms, the graphs include many overlapping points.

#### 3.1.1. Best Candidate Signal Feature for OHS Detection

The Vicon reference measurements suggested that OHS occurred around 550 ms after HS. Comparison of the median difference between the two systems’ measurements for all candidates showed that *F_gx1_* had the best synchronicity with the reference OHS value, with a median difference of 0 ms. However, according to the Wilcoxon signed-rank test, the null hypothesis that the median value was 0 was rejected (*p* < 0.001). This result indicated that there was still a fixed bias between *F_gx1_* and the reference OHS value. Additionally, we compared the precisions between the two sets of measurements in terms of the QD and Kendall’s W. From this comparison, *F_gx1_* had the best performance for OHS detection (better than *F_z1_* and *F_y1_*), with the best Kendall’s W value of 0.966. This result suggested almost perfect agreement when using this feature to detect OHS. The *F_gx1_* were the concave GTPs in a monotonically increasing waveform that appeared after heel rise in *G_x_*, which suggested that, at the moment of OHS, the foot rotation shifted from a low angular acceleration to a high angular acceleration in the plantarflexion direction. According to the Spearman’s correlation analysis for the Bland–Altman plots, there was no correlation between the averages and differences of the two systems’ measurements (*r* = −0.022, *p* = 0.614). Therefore, there was no proportional bias between the two systems. 

#### 3.1.2. Best Candidate Signal Feature for OTO Detection

The Vicon reference measurements suggested that OTO occurred around 120 ms after HS. Comparison of the median difference between the two systems’ measurements for all candidates showed that the feature *F_gx2_* + 2% GC most closely approached the reference OTO value, with a median difference of 0 ms. Moreover, the Wilcoxon signed-rank test suggested that there was no fixed bias between the two systems (*p* = 0.151). Additionally, the Kendall’s W of *F_gx2_* + 2% GC was 0.837, the best value among the three candidates, which suggested strong agreement. Accordingly, this feature was considered the most suitable for OTO detection. Specifically, it indicated that OTO occurs at 2% GC after the foot ends its rotation motion and has landed completely. According to the Spearman’s correlation analysis for the Bland–Altman plots, there was a correlation between the averages and differences of the two systems’ measurements (*r* = −0.372, *p* < 0.001). Moreover, the Bland–Altman plots showed a tiny proportional bias between the two systems, such that when the average became larger, the difference became larger in the negative direction. This result suggested that when the average becomes larger, the IMS value of OTO becomes earlier than the reference OTO value.

### 3.2. Evaluation of Using GPBLL Measurements for Gait Event Detection

#### 3.2.1. Results for One-Stride Measurement

**Figure 11:**
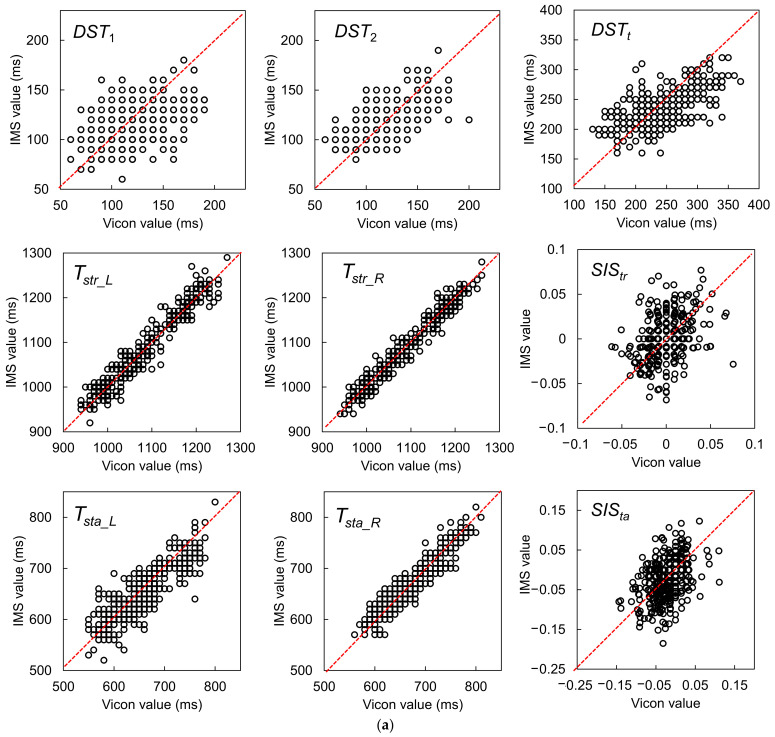

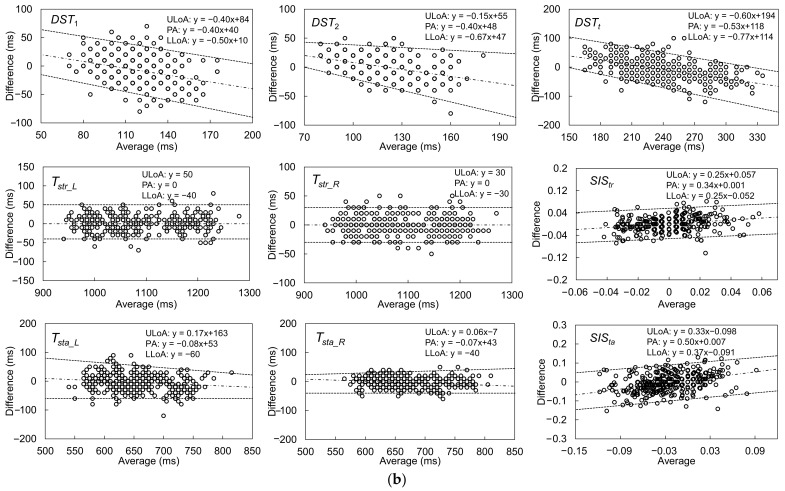
(**a**) Agreement plots between the IMS and Vicon results for gait parameters measured from one stride, including *DST*_1_, *DST*_2_, *DST_t_*, *T_str_L_*, *T_str_R_*, *SIS_tr_*, *T_sta_L_*, *T_sta_R_*, and *SIS_ta_*. (**b**) Bland–Altman plots and LoAs of the 95% confidence interval around the PA line for comparison of the one-stride IMS and Vicon gait parameter measurement results. The black dashed lines are the PA lines, and each pair of black dotted lines represents the ULoA and LLoA. The LoAs of the parameters judged as having a proportional bias were corrected by a nonparametric approach via quantile regression.

The results of the GPBLL measurements from one stride are listed in Table 2. All of these GPBLLs were judged as not following a normal distribution. As a result, instead of using the average and SD, we used the median and quartiles to represent the data distribution, and the median and QD of the differences between the two systems’ results to represent the accuracy and precision, respectively. Figure 11a,b show the agreement plots and Bland–Altman plots, respectively. The LoAs of the parameters that were judged as having a proportional bias were corrected by a nonparametric approach via quantile regression.

The Kendall’s W values of the GPBLLs suggest that the IMS measurements of all these parameters had strong agreement with the Vicon measurements. In particular, *T_str_L_*, *T_str_R_*, *T_sta_L_*, and *T_sta_R_* had very high values near the “perfect agreement” level. All the temporal parameters had precisions no higher than 25 ms, i.e., a deviation of no more than 3 units of 100 Hz measurement, although some of them had a slight proportional bias. Moreover, the precisions of the two symmetry indexes, *SIS_tr_* and *SIS_ta_*, were 0.014 and 0.032, respectively. The Wilcoxon signed-rank test showed no fixed biases in *DST*_2_, *T_str_L_*, *T_str_R_*, *SIS_tr_*, and *SIS_ta_*, and the Spearman’s correlation test showed no proportional biases in *T_str_L_* and *T_str_R_*.

#### 3.2.2. Results for Multiple-Stride Measurement

**Figure 12:**
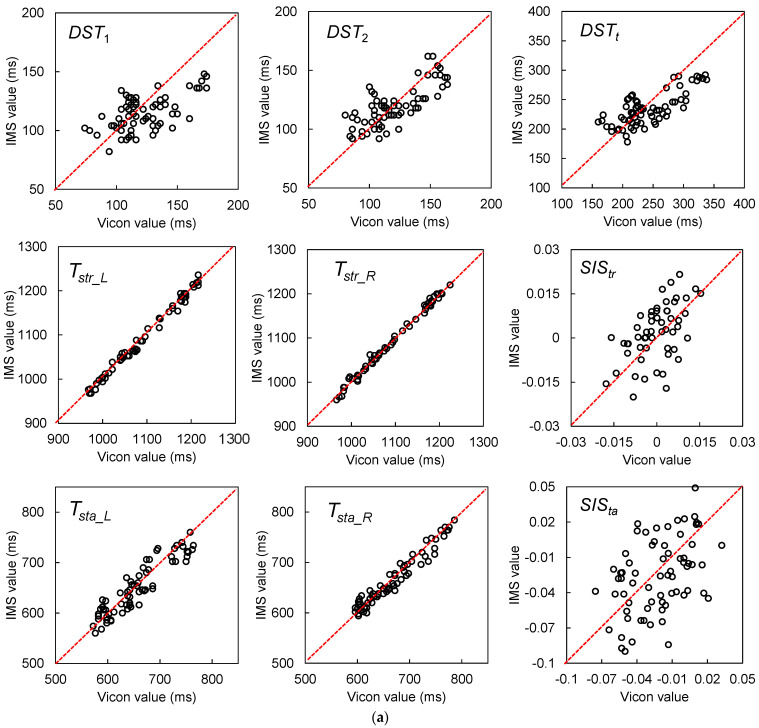

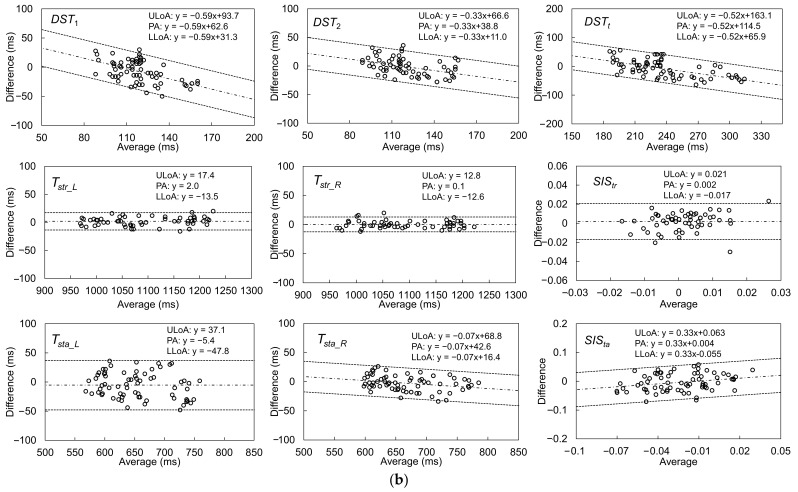
(**a**) Agreement plots between the IMS and Vicon results for gait parameters measured from five continuous effective strides, including *DST*_1_, *DST*_2_, *DST_t_*, *T_str_L_*, *T_str_R_*, *SIS_tr_*, *T_sta_L_*, *T_sta_R_*, and *SIS_ta_*. (**b**) Bland–Altman plots and LoAs of the 95% confidence interval around the PA line for comparison of the multiple-stride IMS and Vicon gait parameter measurement results. The black dashed lines are the PA lines, and each pair of black dotted lines represents the ULoA and LLoA. The LoAs of the parameters judged as having a proportional bias were corrected by a parametric approach via linear regression.

Because walking a distance of 5 m is one of the suggested criteria for an assessment of walking [37], the results of the GPBLL measurements from the average of five continuous effective strides are listed in Table 3. Because all of these GPBLLs were judged as normally distributed, we could use the average and SD to represent the data distribution, and to represent the accuracy and precision, respectively, for the differences between the two systems’ results. Figure 12a,b show the agreement plots and Bland–Altman plots, respectively. The LoAs of the parameters that were judged as having a proportional bias were corrected by a parametric approach via linear regression.

The ICC(2, *k*) values of the five-stride GPBLLs suggest that the IMS measurements of all these parameters had good or excellent agreement with the Vicon measurements. In particular, *DST*_2_, *DST_t_*, *T_str_L_*, *T_str_R_*, *T_sta_L_*, and *T_sta_R_* had excellent agreement. Although there were fixed biases for *DST*_1_, *DST_t_*, and *T_sta_R_*, the precisions of all the temporal parameters were no higher than 10 ms, i.e., a deviation of less than 1 unit of 100 Hz measurement; accordingly, we concluded that there were no fixed biases under the condition of 100 Hz measurement. In contrast, there were still proportional biases for *DST*_1_, *DST*_2_, *DST_t_*, *T_sta_R_*, and *SIS_ta_*. Moreover, the precisions of the two symmetry indexes, *SIS_tr_* and *SIS_ta_*, were 0.010 and 0.030, respectively. Comparison of the SD with the QD suggests that there was no obvious improvement in precision from averaging five continuous effective strides; however, the Bland–Altman plots show that the LoA ranges of *SIS_tr_* and *SIS_ta_* became narrower, which means that their precision was improved through averaging.

## 4. Discussion

In this study, we focused on the basic concept of temporal gait parameters that combine different gait events, which are significant points in a gait cycle. Through foot motion analysis based on biomechanical knowledge, the OHS and OTO can be determined from IMS signals by simple algorithms that are feasible for installation on an edge device such as the IMS in this study. Such OHS and OTO detection enables monitoring of the conditions of both feet with a sensor mounted only on a single foot. 

Nevertheless, some aspects of our results require discussion, as follows. First, the agreement test results described in Section 3.2 demonstrated the reliability of GPBLL measurement by our method. Kirtley indicated that the DST correlates with the walking velocity [38]. It would thus be possible to construct a linear regression model based on the correlation between the walking velocity and the DST from reference data. However, such a constructed model would depend on the data distribution and be sensitive to individual differences. In contrast, our method can detect musculoskeletal status changes during walking, which better reflects the essence of human walking and makes our method more robust against individual differences. Furthermore, the detection and combination of different gait events makes various gait parameters available on the same device and enables them to be evaluated on the same scale.

Here, we acknowledge that this study was limited in that the data were collected only from healthy participants whose walking motions were nearly symmetric. If a walking motion is ideally symmetric, the OHS and OTO should be located halfway between two HSs and two TOs, respectively. Here, we denote the timestamps of these positions as *T_HS_half_* and *T_TO_half_*. Because the OHS and OTO of a healthy person are very near these halfway points, it may be doubtful that the best candidate IMS signal features for OHS and OTO detection, as described above, may have only matched *T_HS_half_* and *T_TO_half_* occasionally, rather than representing the true OHS and OTO points. 

In actual cases, even a healthy young person’s walking motion should have asymmetry [39], such that the OTO and OHS should more or less deviate from the halfway points between two TOs and HSs. In this study, *F_gx1_* had a median of 560 ms and an upper-to-lower quartile range of 520–590 ms, whereas the reference *T_HS_half_* had a median of 540 ms with an upper-to-lower quartile range of 510–580 ms. Similarly, *F_gx2_* had a median of 120 ms with an upper-to-lower quartile range of 110–130 ms, whereas the reference *T_TO_half_* had a median of 130 ms with an upper-to-lower quartile range of 110–150 ms. According to the Wilcoxon signed-rank test, the null hypotheses that the IMS system’s measured OHS and OTO timings were similar to *T_HS_half_* and *T_TO_half_*, respectively, were both rejected (*p* < 0.001, *p* < 0.001), which means that our proposed features from IMS signals did not represent *T_HS_half_* and *T_TO_half_*.

By comparison, according to the agreement tests, *F_gx1_* and *F_gx1_* + 2% GC had Kendall’s W values of 0.966 and 0.837, respectively. Moreover, the Kendall’s W of 0.602 for *SIS_ta_*, which relied on the precision of both the OTO and OHS measurements, suggested that even if only weak asymmetries were found in the healthy participants, they were still evaluated well. Accordingly, we conclude that our proposed feature points could capture changes in the participants’ gait asymmetry well, which further suggests that the proposed method is reliable for detection of OHS and OTO events from IMS signals. Nevertheless, the method still requires testing on participants whose gait is obviously asymmetric.

Table 4 lists the other examples of daily healthcare or activity monitoring applications for users without neurological/orthopedic problems, references to modern studies of those applications [10,12,40,41,42], and the required estimation precisions. The estimation precisions that we obtained in five-stride measurements suggest that the proposed method may have potential for those applications, such as fall prediction, the detection of fatigue, obesity, mild cognitive impairment, or depression. 

OHS detection strongly depends on the knee flexion that passively results from the combined motion of hip flexion and ankle plantarflexion before TO, and OTO detection strongly depends on dorsiflexion motion and the heel rocker function [17,28]. However, if we expand our method to medical applications in the future, we must address another issue: the substantial motions required for OHS and OTO detection are significantly weakened in subjects with ankle osteoarthritis [43], subjects with drop foot induced by tibialis anterior and fibula muscle weakness [44], and hemiplegic subjects [45]. Accordingly, our method may be difficult to apply in such cases, or it may show a significant decrease in precision. Further clinical studies will thus be necessary to validate the applicability of our method in medical applications.

## 5. Conclusions

In this study, we successfully established a method for estimating temporal GPBLLs of healthy subjects by detecting OHS and OTO from foot-motion signals. 

OHS can be determined by searching for a feature consisting of a concave GTP in the monotonically increasing waveform for the *G_x_* signal, which appears after a foot-flat state, with a precision of 15 ms. OTO can be specified by a concave GTP induced by the *G_x_* waveform returning to zero after HS in one GC, with a precision of 10 ms. 

By averaging over five successive effective strides, an IMS can estimate all GPBLLs and achieve “good” or “excellent” agreement with the measurements from a reference Vicon system. All time parameters achieved precisions of no more than 30 ms, and the symmetry indexes of the stride time and stance phase time achieved precisions of 1.0% and 3.0%, respectively. These results suggest that our method shows promise for expanding the potential use of IMSs in daily healthcare or activity monitoring applications with the target to improve habits for healthy subjects. Furthermore, because the proposed method can detect motion of the opposite foot, our method may enable monitoring of the conditions of both feet with a sensor worn only on one foot.

In the future, we will test the applicability of the proposed method for subjects with musculoskeletal ambulation problems, older subjects, and subjects whose gaits are obviously asymmetric.

## Figures and Tables

**Table 1:** Summary of the synchronicity evaluation results for candidate features.

Gait Event	Feature	Relative Time to HS (ms)		Temporal Difference (ms)				Kendall’s W
		Vicon (Median (Lower–Upper Quartile))	IMS (Median (Lower–Upper Quartile))	Accuracy (Median)	Precision (QD)	Fixed Bias? (*p*-Value)	Proportional Bias? (*r*, *p*-Value)	
OHS	*F_y1_*	550 (520–590)	620 (570–670)	60	20	*p* < 0.001	*r* = 0.279, *p* < 0.001	0.943
	*F_z1_*		570 (530–605)	10	15	*p* < 0.001	*r* = 0.095, *p* = 0.028	0.963
	***F_gx1_***		560 (520–590)	0	15	*p* < 0.001	*r* = −0.022, *p* = 0.614	0.966
OTO	*F_y2_* + 2%GC	120 (100–140)	110 (90–120)	−10	20	*p* < 0.001	*r* = −0.049, *p* = 0.250	0.618
	*F_z2_* + 2%GC		110 (100–130)	−10	20	*p* < 0.001	*r* = −0.589, *p* < 0.001	0.727
	***F_gx2_* + 2%GC**		120 (110–130)	0	10	*p* = 0.151	*r* = −0.372, *p* < 0.001	0.837

**Table 2:** Summary of the synchronicity evaluation results for candidate features.

Gait Parameter	Median (Lower–Upper Quartile)		Temporal Difference			Kendall’s W
	Vicon (ms)	IMS (ms)	Accuracy and Precision (Median (QD), ms)	Fixed Bias? (*p*-Value)	Proportional Bias? (*r*, *p*-Value)	
*DST*_1_ (ms)	120 (107.5–140)	110 (100–130)	−10 (15)	Y *p* < 0.001	*r* = −0.279, *p* < 0.001	0.687
*DST*_2_ (ms)	120 (110–140)	120 (110–130)	0 (10)	N *p* = 0.608	*r* = −0.410, *p* < 0.001	0.814
*DST_t_* (ms)	240 (210–270)	230 (210–250)	0 (25)	Y *p* < 0.001	*r* = −0.485, *p* < 0.001	0.794
*T_str_L_* (ms)	1080 (1020–1170)	1070 (1020–1160)	0 (10)	N *p* = 0.529	*r* = 0.033, *p* = 0.549	0.985
*T_str_R_* (ms)	1070 (1020–1160)	1070 (1020–1160)	0 (10)	N *p* = 0.612	*r* = 0.069, *p* = 0.206	0.988
*T_sta_L_* (ms)	650 (600–690)	640 (610–690)	0 (20)	Y *p* = 0.032	*r* = −0.182, *p* < 0.001	0.925
*T_sta_R_* (ms)	665 (620–710)	665 (620–700)	0 (15)	Y *p* = 0.022	*r* = −0.172, *p* < 0.001	0.967
*SIS_tr_*	0.000 (−0.011–0.010)	0.000 (−0.017–0.017)	0.000 (0.014)	N *p* = 0.379	*r* = 0.250, *p* < 0.001	0.698
*SIS_ta_*	−0.017 (−0.046–0.000)	−0.026 (−0.060–0.014)	0.000 (0.032)	N *p* = 0.708	*r* = 0.359, *p* < 0.001	0.708

**Table 3:** Accuracy and precision evaluation results for temporal gait parameters obtained by averaging five strides.

Gait Parameter	Accuracy (Average, ms)	Precision (SD, ms)	Fixed Bias? (*p*-Value)	Proportional Bias? (*r*, *p*-Value)	ICC(2,*k*)
*DST*_1_ (ms)	−6.9	18.9	Y *p* = 0.003	*r* = −0.530, *p* = <0.001	0.665
*DST*_2_ (ms)	−1.3	15.4	N *p* = 0.493	*r* = −0.410, *p* = <0.001	0.835
*DST_t_* (ms)	−8.2	30.1	Y *p* = 0.027	*r* = −0.596, *p* = <0.001	0.800
*T_str_L_* (ms)	2.0	8.0	N *p* = 0.707	*r* = 0.160, *p* = 0.238	0.998
*T_str_R_* (ms)	0.1	6.5	N *p* = 0.935	*r* = −0.024, *p* = 0.864	0.998
*T_sta_L_* (ms)	−5.4	21.8	Y *p* = 0.045	*r* = −0.216, *p* = 0.075	0.957
*T_sta_R_* (ms)	−2.8	13.3	N *p* = 0.081	*r* = −0.273, *p* = 0.023	0.984
*SIS_tr_*	0.002	0.010	N *p* = 0.186	*r* = 0.250, *p* = 0.063	0.654
*SIS_ta_*	−0.004	0.030	N *p* = 0.297	*r* = 0.263, *p* = 0.029	0.602

**Table 4:** Potential applications and required estimation precisions for gait parameters measured by an IMS.

Potential Application	Gait Parameter	Required Precision	Achieved Precision	Reference
Fall	*DST_total_*	80 ms	18.9 ms	[10]
Fatigue	*DST_total_*	2.00%GC or 17–26 ms	18.9 ms	[12]
Obesity	*T_sta_L_* or *T_sta_R_*	1.70%GC or 15–24 ms	8.0 or 6.5 ms	[40]
	*DST_total_*	3.48%GC or 30–45 ms	18.9 ms	
Mild cognitive impairment	*DST_total_*	30 ms	18.9 ms	[41]
Depression	*DST_total_*	54 ms	18.9 ms	[42]
	*T_sta_L_* or *T_sta_L_*	65 ms	8.0 or 6.5 ms	

## Supplementary Materials

The following are available online at https://www.mdpi.com/article/10.3390/s22010351/s1, Figure S1: Synchronization of the Vicon and IMS *E_x_* waveform signals.
