# Supplementary material for: Method for Estimating Temporal Gait Parameters Concerning Bilateral Lower Limbs of Healthy Subjects Using a Single In-Shoe Motion Sensor through a Gait Event Detection Approach
Source: Sensors (Basel). 2022 Jan 4;22(1):351. doi: 10.3390/s22010351 (PMC8749800; doi:10.3390/s22010351)
Supplement: Supplementary file 1 [file sensors-22-00351-s001.zip › sensors-1512771-supplementary.pdf]

sensors

MDPI

Supplementary Materials

Supplementary Materials

Chenhui Huang *, Kenichiro Fukushi, Zhenwei Wang, Fumiyuki Nihey, Hiroshi Kajitani, Kentaro Nakahara

Biometrics Research Labs, NEC Corporation, Hinode 1131, Abiko, Chiba 270-1174, Japan
* Correspondence: chenhui.huang@nec.com; Tel.: +81 08088185507

The synchronization of reference system Vicon and in-shoe motion sensor (IMS)

A 3D motion analysis system, the Track 3 (Vicon Motion Systems, UK), was used to obtain reference measurements for evaluate the proposed estimation method for temporal gait parameters concerning bilateral lower limbs of healthy subjects using single in-Shoe motion sensor. To find which candidate point is the best one for OHS and OTO detection and for the evaluation of the proposed GPBLLs estimation method, we first needed to completely synchronize the signals acquired by the Vicon system and the IMS, we first needed to completely synchronize the signals acquired by the Vicon system and the IMS.

Because they operated independently, their data collection starting times were not simultaneous. In our study, there was no hardware connection between the two systems. Regarding the definition of axis direction, the rotation of the X-axis here means the alteration of the sole-to-ground angle in the dorsal-flexion/plantar-flexion (E_x) direction. It was found that the waveform of E_x waveform signals at the beginning of a walking trial [shown in Fig. S1 in red dashed circles] was different from white noise/random motion and walking at a uniform velocity. Thus, this part of the waveform could be utilized for synchronizing the two signals acquired from the different systems. Therefore, instead, as shown in Fig. 5, a software synchronization method was applied to superpose the E_x waveform signals taken by the different instruments in each walking trial.

First, a window was set on this particular part of the IMS waveform. Then, it was shifted on the temporal axis until it was completely superposed on the same part of the Vicon waveform. The superposing position was judged on the basis of the appearance of the maximum Pearson product-moment correlation coefficient between the two waveforms. Then the timestamps of the waveform whose length was longer were corrected according to the length of the shorter one to assure they can superpose each other well on the same temporal axis.

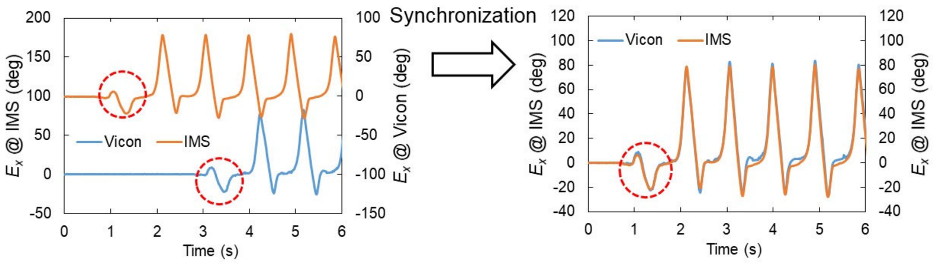

Figure S1. Synchronization of the Vicon and IMS E_x waveform signals.
